# Pitfalls of commercially available HPV tests in HPV68a detection

**DOI:** 10.1371/journal.pone.0220373

**Published:** 2019-08-05

**Authors:** Hana Jaworek, Katerina Kubanova, Vladimira Koudelakova, Rastislav Slavkovsky, Jiri Drabek, Marian Hajduch

**Affiliations:** 1 Institute of Molecular and Translational Medicine, Faculty of Medicine and Dentistry, Palacky University Olomouc, Olomouc, Czech Republic; 2 Cancer Research Czech Republic, Olomouc, Czech Republic; Penn State University School of Medicine, UNITED STATES

## Abstract

**Background:**

Human papillomavirus 68 (HPV68) is a probable carcinogenic HPV genotype which is included in almost all HPV screening assays and exists as two genetically variable subtypes (HPV68a and HPV68b). Routine HPV sample testing has shown that the cobas 4800 HPV Test (Roche) provides higher false-negative rates for HPV68 status than PapilloCheck HPV-Screening (Greiner Bio-One). The aim of our study was to evaluate the efficacy of cobas 4800 in HPV68 detection.

**Methods:**

A total of 2,145 cervical/cervicovaginal samples from women aged 17–88 were tested for HPV68 status using the cobas 4800 and PapilloCheck HPV tests. Viral load was assessed by quantitative PCR in all of the HPV68-positive cases. HPV68a/b subtyping was performed with real-time PCR followed by high resolution melting curve analysis, and was subsequently confirmed by Sanger sequencing.

**Results:**

Cobas 4800 detected HPV positivity in only 13/33 HPV68 single-genotype infection cases. Viral load was comparable across both tested subgroups. HRM analysis and Sanger sequencing identified the HPV68a subtype in all of the 20 instances of cobas 4800 false negatives. HPV68a and HPV68b were detected in 3/13 and 10/13 cases identified as other HPV-positive by cobas 4800.

**Conclusion:**

The HPV68a subtype was missed by cobas 4800 in more than 85% of all HPV68a-positive cases. Therefore, commercially available assays may underestimate HPV68 prevalence.

## Introduction

Cervical cancer is caused by persistent infection by at least one high-risk human papillomavirus (hrHPV) genotype [1–3]. The high-risk HPV genotypes HPV16 and HPV18 are associated with 70% of cervical cancer cases worldwide, while most of the remaining cases are associated with other hrHPV genotypes including HPV31, 33, 35, 39, 45, 51, 52, 56, 58, 59, 66, 68, 73 and 82 [1–3].

HPV68, a probable carcinogenic agent, is found in less than 1% of cervical cancers [4]; however, the prevalence of HPV68 and other non-targeted genotypes may increase following increasing vaccination by a nonavalent HPV vaccine targeting HPV6, 11, 16, 18, 31, 33, 45, 52 and 58 due to the selection pressure [5]. HPV68 may exist in two subtypes (a and b), in which the E6, E7 and L1 open reading frame (ORF) sequences differ by 6%, 5% and 7%, respectively [6]. Previous research has shown that the HPV68a subtype is insufficiently amplified when using an assay based on the well-established PGMY primer set to target the L1 ORF [7]. Even though several PGMY-based assays have updated the primer sets and reported improved HPV68 coverage [8], a majority of PGMY-based assays still only reliably detect HPV68b [9]. The cobas 4800 HPV Test (Roche Diagnostics, Mannheim, Germany; herein referred to as “cobas 4800”) is a widely used real-time PCR-based assay targeting the L1 gene. This assay provides full HPV16 and HPV18 genotyping in addition to pooling the results of 12 other hrHPV genotypes, including HPV68 [10;11].

Our laboratory routinely uses two HPV detection systems—cobas 4800 with partial genotyping and PapilloCheck HPV-Screening (Greiner Bio-One, Frickenhausen, Germany; herein “PapilloCheck”) with full genotyping. Surprisingly, more than half of the HPV68-positive cases detected by PapilloCheck were classified as HPV-negative by cobas 4800. As such, the aim of our study was to evaluate the efficacy of the routinely used cobas 4800 HPV Test in detecting the HPV68 genotype.

## Material and methods

### Ethical considerations

This study was performed in compliance with the Helsinki Declaration and the research proposal was approved by the Ethics Committees of both the Faculty of Medicine and Dentistry at Palacky University and the Faculty Hospital in Olomouc (approval number 29/13). Written informed consent regarding the use of collected samples for research was obtained from all study participants.

### Clinical specimens’ collection

For this study, 2,415 samples were collected between February 2013 and June 2016 from Czech women aged 17–88 (median age 33) regardless of histopathology or cytomorphology findings. All of the samples– 2,198 cervical swabs and 217 self-sampled cervicovaginal swabs—were stored in cobas PCR Cell Collection Media (Roche Diagnostics, Risch-Rotkreuz, Switzerland).

The self-sampled cervicovaginal swabs, which were collected using the Evalyn Brush device (Rovers Medical Devices B.V., Oss, Netherlands), were part of a cervical cancer prevention program organized by charity Cancer Research Czech Republic [12].

### HPV DNA detection

**Fig 1:**
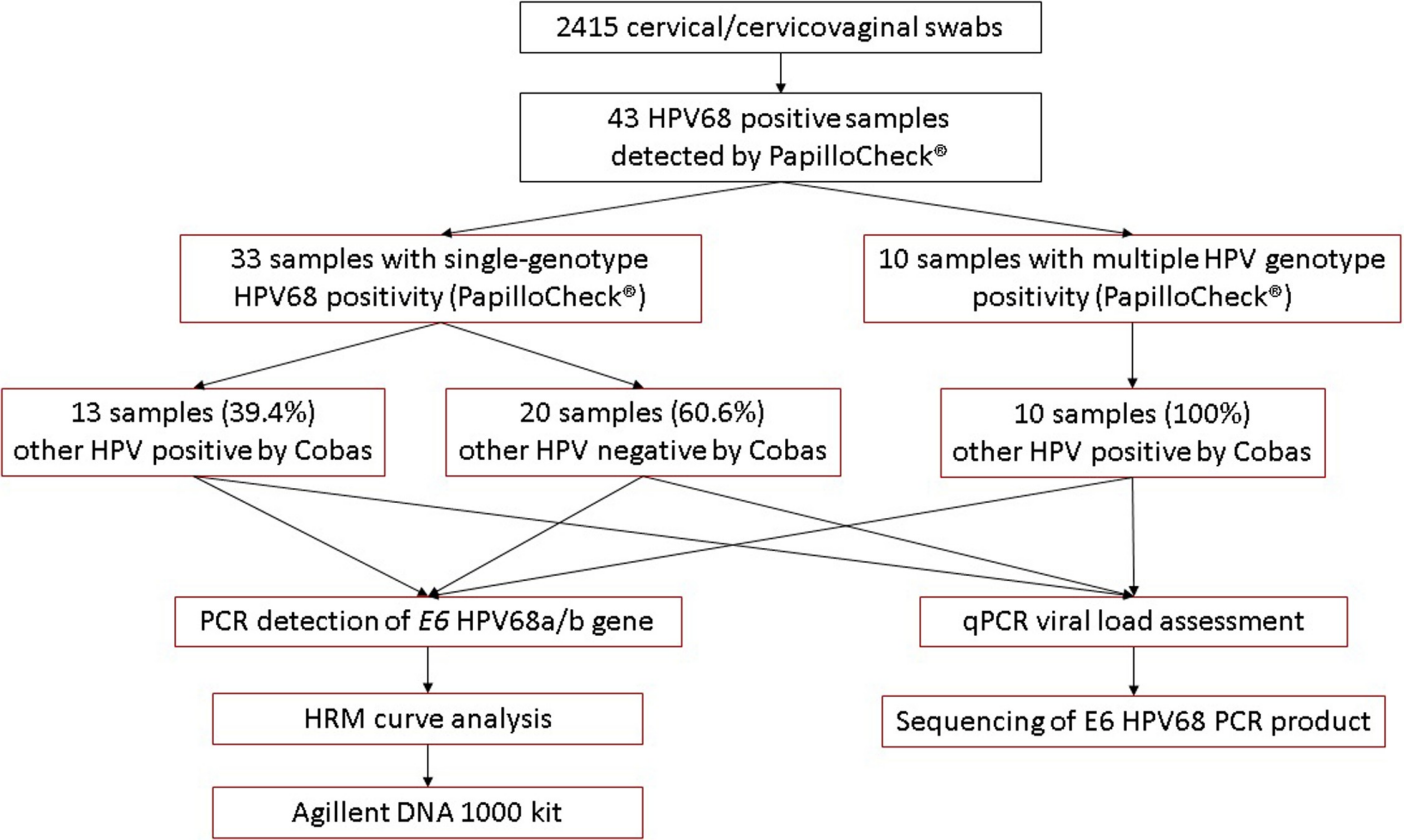
Study flowchart.

All of the collected samples were tested for HPV DNA using the cobas 4800 HPV Test (Roche Diagnostics) according to the manufacturer’s recommendations. Cobas 4800 separately detects HPV16, 18 and 12 other hrHPV genotypes (HPV31, 33, 35, 39, 45, 51, 52, 56, 58, 59, 66, and 68) in a pooled result (referred to as “other HPV positive”) [10]. DNA extracted using the cobas x 480 automated instrument (Roche Diagnostics) was also subjected to HPV DNA detection using E1-targeting PapilloCheck HPV-Screening (Greiner Bio-One, Kremsmünster, Austria; herein “PapilloCheck”). PapilloCheck provides full genotyping information about 18 hrHPV (HPV16, 18, 31, 33, 35, 39, 45, 51, 52, 53, 56, 58, 59, 66, 68, 70, 73, and 82) and 6 lrHPV genotypes (HPV6, 11, 40, 42, 43, and 44/55) [13]. The application of DNA extracted by cobas x 480 for PapilloCheck was validated earlier [14]. Samples were divided into three groups according to the results of PapilloCheck and cobas 4800 assays (Fig 1):

1. other HPV-positive by cobas 4800, HPV68-positive by PapilloCheck (N = 13)
2. other HPV-positive by cobas 4800, HPV68-positive and other hrHPV-positive by PapilloCheck (N = 10)
3. other HPV-negative by cobas 4800, HPV68-positive by PapilloCheck (N = 20)

Both tests were repeated in the case of discordant results. All of the 43 HPV68-positive samples were subjected to further analyses of viral load and presence of HPV68 subtypes (Fig 1).

### Viral load assessment

HPV68 viral load was evaluated by multiplex quantitative real-time polymerase chain reaction (qPCR) targeting the E6 HPV gene and human GAPDH (glyceraldehyde-3-phosphate dehydrogenase) genes. GAPDH detection served as an internal control of amplification. Probes and primers were designed using the Primer3 software [15]. Sequence specificity of all oligonucleotides was evaluated using BLAST (BLAST, National Center for Biotechnology Information, Bethesda, MD). The PCR primers and probes listed in Table 1 were synthesized by Generi Biotech Ltd. (Hradec Kralove, the Czech Republic). The qPCR analysis was performed using a Light Cycler 480 II (Roche Diagnostics, Mannheim, Germany) according to a previously described protocol [16].

**Table 1:** Characteristics of HPV68 E6 primers/probes.

gene	primer/probe	DNA sequence	product size (bp)
HPV68 E6*	Forward	5′-CCGTGCAGGAAATTGTGTTAG-3′	96
	Reverse	5′-TTTCATCGTCTGAATCTCCTAATTG-3′	
	Probe	BHQ1-TCGTGACATACAAGGTCAACCGGC-HEX	
GAPDH*	Forward	5′-GAGTGAGTGGAAGACAGAATG-3′	70
	Reverse	5′-CAACTAGGATGGTGTGGCTCCC-3′	
	Probe	BHQ1-GGGACACAAGGTTACCATATAC-CY5	
HPV68a E6**	Forward	5′-GAAAAACTAAGGCACCTAA-3′	56
HPV68b E6**	Forward	5′-CACCTAAATTCAAAACGAAGAATTC-3′	44
HPV68a/b E6**	Reverse	5′-CATAAAATAGCAGGAAACTTT-3′	

BHQ1, Black Hole Quencher 1; CY5, cyanine5; HEX, hexachloro-fluorescein

* primers and probes used for multiplex qPCR (viral load assessment)

** primers used for PCR with high resolution melting analysis

The qPCR limit of detection (LOD) was assessed using a dilution series of plasmid containing HPV68 DNA (HPV68 clone in pBScript vector; kindly provided by Carina Eklund; Karolinska Institutet, Stockholm, Sweden) and HPV68 DNA plasmid coupled with 50 ng of DNA isolated from CCRF-CEM cell line (ATCC, Rockwille, MD). Dilution series of 4, 40, 400, 4,000, 40,000 and 400,000 copies of a HPV68 clone per reaction were analyzed in duplicates over five technical replicates.

HPV68 viral load was expressed as HPV *E6* gene copy number per ng of DNA. DNA concentration was measured using the fluorescence-based Qubit dsDNA HS Assay Kit (Thermo Fisher Scientific, Waltham, MA).

### HPV68 subtyping

Real-time PCR followed by high resolution melting (HRM) curve analysis was used to distinguish the presence of HPV68a and HPV68b subtypes. Primers were designed using uMELT software [17]. The oligonucleotides listed in Table 1 were synthesized by Generi Biotech Ltd. (Hradec Kralove, the Czech Republic). The HPV68a and HPV68b E6 gene fragments that were used as standards had been synthetized by Thermo Fisher Scientific.

PCR was performed in a 25 μl reaction volume containing 2x HotStarTaq Plus Master Mix (Qiagen, Hilden, Germany), 0.5x EvaGreen Dye (Biotium, Fremont, CA), 125 nM of each primer, and 2 μl of DNA.

Amplification and detection was performed using a Light Cycler 480 II. The PCR conditions were 95°C for 15 minutes, which was followed by 10 cycles of 95°C for 10 seconds, 66°C for 20 seconds and 72°C for 20 seconds, and a further 45 cycles of 95°C for 10 seconds, 58°C for 20 seconds and 72°C for 20 seconds. In the HRM analysis, PCR products were heated to 95°C for 70 seconds then cooled to 60°C for 45 seconds. The melting step of the HRM analysis proceeded at an increment of 0.06°C/s from 60°C to 95°C. The decrease in fluorescence was measured simultaneously. LightCycler480 software was used for data evaluation. The melting temperatures (Tm) of HPV68a and HPV68b PCR products were determined to be 73°C and 69°C, respectively.

The HRM analysis results were confirmed by an analysis of the HPV68a and HPV68b PCR products using the Agilent DNA 1000 kit (Agilent Technologies, Santa Clara, CA). HPV68 subtyping was also verified by sequencing the E6 PCR products obtained by qPCR for viral load assessment. The Sanger sequencing was performed by SEQme Ltd. (Dobris, the Czech Republic).

## Results

### HPV68 DNA detection system

HPV68 was detected in 39 out of 2,198 (1.77%) cervical swabs and 4 out of 217 (1.84%) cervicovaginal swabs when PapilloCheck was used. Out of the 43 HPV68-positive samples, 33 samples were positive for HPV68 single-genotype infection. Cobas 4800 did not detect HPV positivity in 60.6% (20/33) of these cases (Fig 1). For this reason, another method for confirming HPV68 positivity was designed and validated.

The LOD of the HPV68-specific quantitative multiplex PCR was determined using a dilution series of plasmid DNA containing the HPV68 genome with or without genomic DNA isolated from CCRF-CEM cell line. The dilution series—which ranged from 4 to 400,000 copies of HPV genome per reaction—was analyzed over five technical replicates. The qPCR method was able to reliably detect HPV68 in samples that contained at least 40 copies of the HPV68 *E6* gene regardless of genomic DNA presence (Table 2). Samples were also tested for cross-reactivity with other HPV genotypes. HPV68-specific qPCR confirmed HPV68 positivity in all 43 samples that had tested positive for HPV68 using PapilloCheck.

**Table 2:** Evaluation of the detection limit of the qPCR method.

copies of HPV/reaction	HPV68* (95% CI)	HPV68*^#^ (95% CI)
4x10^5^	19.62 (19.61–19.64)	19.53 (19.50–19.55)
4x10^4^	22.95 (22.91–22.98)	22.90 (22.86–22.98)
4x10^3^	26.15 (26.12–26.17)	26.26 (26.21–26.29)
4x10^2^	30.16 (30.14–30.21)	30.75 (30.60–30.97)
4x10^1^	35.26 (34.98–35.58)	36.32 (36.07–36.67)
4x10^0^	37.31 (36.84–38.02)	37.69 (37.07–37.81)

CI, confidence interval

*The average C_T_ value of 5 dilution series analysed in duplicate.

^#^50 ng of DNA isolated from CCRF-CEM cell line added per reaction

### Viral load assessment

HPV68-specific qPCR was also used to exclude the effect of viral load on the efficacy at which cobas 4800 identifies HPV positivity/negativity. Thirteen of the 33 HPV68-single genotype positive samples (39.4%) were identified as other HPV-positive by cobas 4800. In these samples, the median viral load was 281 E6 copies/ng DNA, with a range of 9 to 17229 E6 copies/ng DNA. Twenty of the 33 HPV68-single genotype positive samples (60.6%) were identified as HPV-negative by cobas 4800. The median viral load in these samples was 1548 E6 copies/ng DNA, with a range of 1 to 320175 E6 copies/ng DNA (Table 3).

**Table 3:** The distribution of HPV68 positivity as identified by the cobas 4800 HPV Test and PapilloCheck HPV-Screening test. The median viral loads, as measured by qPCR, and ratios of HPV68a/b subtypes, determined through high resolution melting analysis and DNA sequencing, are shown for each HPV result combination.

cobas 4800 HPV Test result	PapilloCheck HPV-Screening test result	N	qPCR	PCR and HRM		Sanger sequencing	
			viral load (E6 copies/ng)*	HPV68a	HPV68b	HPV68a	HPV68b
other HPV+	only HPV68+	13	281 (9–17229)	5/13 (38.5%)	8/13 (61.5%)	3/13 (23.1%)	10/13 (76.9%)
other HPV+	HPV68 and other HPV+	10	254 (3–14584)	6/10 (60%)	4/10 (40%)	6/10 (60%)	4/10 (40%)
other HPV-	only HPV68+	20	1548 (1–320175)	20/20 (100%)	0/20 (0%)	20/20 (100%)	0/20 (0%)

N, number of cases; HPV+, human papillomavirus positive; HPV-, human papillomavirus negative; qPCR, quantitative polymerase chain reaction; PCR, polymerase chain reaction; HRM, high resolution melting

* Median viral load value of HPV E6 gene copy number per ng of DNA (range of viral loads).

### HPV68 subtyping

Real-time PCR, followed by a HRM analysis, was used for HPV68a/b subtyping. HPV68a and HPV68b subtypes were detected in 72.1% (31/43) and 27.9% (12/43) of all HPV68-positive cases, respectively. HRM analysis confirmed that 38.5% (5/13) and 61.5% (8/13) of the samples with HPV68 single-type positivity that were classified as other HPV-positive by cobas 4800 included the HPV68a and HPV68b subtypes, respectively. In this subgroup, Sanger sequencing confirmed all of the HPV68b cases and reclassified two samples that had been identified as HPV68a-positive by HRM to actually be HPV68b-positive.

Interestingly, the presence of HPV68a was confirmed by both HRM analysis and DNA sequencing in all 20 cases which were HPV68 single-type positive yet recognized as HPV-negative by cobas 4800 (20/20, 100%) (Tables 3 and S1).

## Discussion

This study aimed to evaluate the efficacy of routinely used hrHPV detection method cobas 4800 HPV Test in HPV68 detection. We found high discrepancy in HPV68 detection between the PapilloCheck and cobas 4800 HPV detection methods during routine sample testing, and for this reason, aimed to find the reason for this observation.

Our results found cobas 4800 to be significantly less sensitive in detecting HPV68a than HPV68b, with cobas 4800 missing 87% (20/23) of HPV68a-positive cases but detecting all HPV68b-positive cases (10/10, 100%). Moreover, both HRM analysis and Sanger sequencing identified the HPV68a subtype in all of the samples that were classified as HPV-negative by cobas 4800 but HPV68-positive by PapilloCheck. It is known that methods using PGMY primer sets targeting L1 gene may only reliably detect the HPV68b subtype and usually miss HPV68a-positive cases. Assays that employ PGMY primer sets include the Linear Array (Roche), CLART HPV2/3 (Genomica), and LCD array (Chipron), among others [9]. Several PGMY-based assays have updated the primer set to improve HPV68 coverage [8]; nevertheless, the majority of PGMY-based assays can still only reliably detect HPV68b [9].

HPV68 is classified as probably carcinogenic (subgroup 2A) by the Working Group of the World Health Organization (WHO) International Agency for Research on Cancer (IARC) [3]; however, several recent studies have proven the carcinogenic potential of HPV68 [4;18;19]. The prevalence of HPV68 in cervical cancer cases—as well as in women with normal cytology—is very low (0.5% in cervical cancer cases and 0.3% in women with normal cytology) relative to other high-risk HPV genotypes [20]. Nevertheless, in our study, the presence of HPV68 genotype was almost four times higher than prevalence what has been reported previously (1.8%; 43/2415). This result may be explained by variations in HPV prevalence according to the region and population studied. Recent studies reported HPV68 to be the most prevalent genotype in the isolated Quilombo community in Brazil. In these studies, HPV68 infection was associated with inflammation rather than cytological abnormalities and was more often observed in multiple genotype infections [21;22]. In contrast, our research found single HPV68 infection to be more common than multiple HPV68 infection (76.7%, 33/43 compared to 23.3%, 10/43).

Previous research may have underestimated HPV68 prevalence because many epidemiologic studies have applied assays targeting the L1 ORF to study the prevalence of different HPV genotypes [23–25]. Another study found the HPV68a subtype to account for 52% (13/25) of HPV68-positive cases. Therefore, L1-targeting assays could miss up to half of all HPV68 cases [8]. In our study, the HPV68a subtype contributed to an even larger proportion (67.4%, 29/43) of HPV68-positive cases.

The low HPV68 prevalence in cervical cancer [20] and substantial loss of screening specificity conferred by adding less carcinogenic HPV genotypes to screening assays [26] could lead to exclusion of HPV68 from newly developed screening tests as well as HPV vaccines. However, it is important to consider that HPV68 prevalence could substantially increase if nonavalent HPV vaccines successfully reduce the prevalence of targeted genotypes. This could cause inefficiencies in the detection and prevention of cervical cancer, as the commonly used L1-targeting assays could miss clinically relevant cases in which women are infected by the HPV68a subtype [1;20].

In conclusion, the cobas 4800 HPV Test was found to have significantly lower sensitivity for the HPV68a than HPV68b subtype, missing more than 85% of HPV68a-positive cases. This finding implies that research which applied L1-targeting assays to study the prevalence of various HPV genotypes may have significantly underestimated HPV68 prevalence.

## Supporting information

S1 Table The distribution of HPV positivity, viral loads, DNA concentrations and HPV68a and HPV68b subtypes in the dataset of 43 cervical/cervicovaginal swabs with HPV68 positivity detected by PapilloCheck HPV-Screening test. (DOCX)
