# Supplementary material for: Pitfalls of commercially available HPV tests in HPV68a detection
Source: PLoS One. 2019 Aug 5;14(8):e0220373. doi: 10.1371/journal.pone.0220373 (PMC6681972; doi:10.1371/journal.pone.0220373)
Supplement: S1 Table — (DOCX) [file pone.0220373.s001.docx]

**S1 Table. The distribution of HPV positivity, viral loads, DNA concentrations and HPV68a and HPV68b subtypes in the dataset of 43 cervical/cervicovaginal swabs with HPV68 positivity detected by PapilloCheck HPV-Screening test.**

|  | cobas 4800 HPV Test result | PapilloCheck HPV-Screening test result | E6  copy number | DNA concentration (ng/ul) | HPV68a/b subtyping using PCR and HRM | Agilent DNA 1000 analysis HPV68a/b subtyping | Sanger sequencing (HPV68a/b) |
| --- | --- | --- | --- | --- | --- | --- | --- |
| 1 | HPV18, other HPV | HPV68 | 12100.00 | 1.23 | HPV68a | HPV68a | HPV68a |
| 2 | other HPV | HPV68 | 858.67 | 0.37 | HPV68b | HPV68b | HPV68b |
| 3 | other HPV | HPV68 | 168.00 | 0.104 | HPV68a | HPV68a | HPV68b |
| 4 | other HPV | HPV68, 53 | 17366.67 | 0.504 | HPV68b | HPV68b | HPV68b |
| 5 | other HPV | HPV68 | 178.33 | 0.49 | HPV68a | HPV68a | HPV68a |
| 6 | other HPV | HPV68 | 567.33 | 1.01 | HPV68b | HPV68b | HPV68b |
| 7 | HPV16, 18, other HPV | HPV16, 18, 68 | 36.70 | 1.33 | HPV68b | HPV68b | HPV68b |
| 8 | other HPV | HPV68 | 12470.00 | 26.6 | HPV68b | HPV68b | HPV68b |
| 9 | other HPV | HPV68 | 304.33 | 1.02 | HPV68b | HPV68b | HPV68b |
| 10 | other HPV | HPV68, 70 | 18600.00 | 1.4 | HPV68a | HPV68a | HPV68a |
| 11 | other HPV | HPV68, 82 | 114000.00 | 18.23 | HPV68b | HPV68b | HPV68b |
| 12 | other HPV | HPV68, 73, 82 | 2216.67 | 4.44 | HPV68b | HPV68b | HPV68b |
| 13 | HPV16, other HPV | HPV16, 68, 42 | 46.70 | 2.54 | HPV68a | HPV68a | HPV68b |
| 14 | other HPV | HPV56, 68, 70 | 583.67 | 0.178 | HPV68a | HPV68a | HPV68a |
| 15 | other HPV | HPV52, 68 | 281.33 | 0.426 | HPV68b | HPV68b | HPV68b |
| 16 | other HPV | HPV52, 68 | 24266.67 | 0.834 | HPV68b | HPV68b | HPV68b |
| 17 | other HPV | HPV 56, 68, 44/55 | 66.00 | 10.1 | HPV68a | HPV68a | HPV68a |
| 18 | other HPV | HPV39, 68, 70 | 7843.33 | 7.94 | HPV68a | HPV68a | HPV68a |
| 19 | other HPV | HPV31, 56, 68 | 46.70 | 0.84 | HPV68a | HPV68a | HPV68a |
| 20 | other HPV | HPV31, 68, 42 | 39200.00 | 110.6 | HPV68a | HPV68a | HPV68a |
| 21 | other HPV | HPV56, 68 | 13633.33 | 46.86 | HPV68a | HPV68a | HPV68a |
| 22 | HPV16, other HPV | HPV16, 56, 68 | 46.70 | 2.187 | HPV68b | HPV68b | HPV68b |
| 23 | other HPV | HPV39, 68, 66, 70 | 38633.33 | 2.547 | HPV68b | HPV68b | HPV68b |
| 24 | negative | HPV68 | 484.67 | 0.198 | HPV68a | HPV68a | HPV68a |
| 25 | negative | HPV68, 70 | 219000.00 | 0.342 | HPV68a | HPV68a | HPV68a |
| 26 | negative | HPV68 | 17266.67 | 2 | HPV68a | HPV68a | HPV68a |
| 27 | negative | HPV68 | 77233.33 | 1.22 | HPV68a | HPV68a | HPV68a |
| 28 | negative | HPV68 | 1946.67 | 0.52 | HPV68a | HPV68a | HPV68a |
| 29 | HPV16 | HPV16, 68 | 1203.33 | 0.656 | HPV68a | HPV68a | HPV68a |
| 30 | negative | HPV68, 42 | 10833.33 | 0.296 | HPV68a | HPV68a | HPV68a |
| 31 | negative | HPV68 | 7453.33 | 111 | HPV68a | HPV68a | HPV68a |
| 32 | negative | HPV68 | 36.70 | 25.6 | HPV68a | HPV68a | HPV68a |
| 33 | negative | HPV68,42 | 2616.67 | 3.81 | HPV68a | HPV68a | HPV68a |
| 34 | negative | HPV68 | 75366.67 | 4.05 | HPV68a | HPV68a | HPV68a |
| 35 | negative | HPV68, 59 | 41100.00 | 2.206 | HPV68a | HPV68a | HPV68a |
| 36 | negative | HPV68 | 156000.00 | 10.16 | HPV68a | HPV68a | HPV68a |
| 37 | negative | HPV68 | 1006.67 | 1.02 | HPV68a | HPV68a | HPV68a |
| 38 | negative | HPV68 | 4500.00 | 3.47 | HPV68a | HPV68a | HPV68a |
| 39 | negative | HPV68 | 46.70 | 2.873 | HPV68a | HPV68a | HPV68a |
| 40 | negative | HPV68, 42 | 270.67 | 2.413 | HPV68a | HPV68a | HPV68a |
| 41 | negative | HPV68, 44/55 | 9460.00 | 2.403 | HPV68a | HPV68a | HPV68a |
| 42 | negative | HPV68 | 126000.00 | 2.597 | HPV68a | HPV68a | HPV68a |
| 43 | negative | HPV68 | 46.70 | 2.48 | HPV68a | HPV68a | HPV68a |

Other HPV includes HPV 31, 33, 35, 39, 45, 51, 52, 56, 58, 59, 66, and 68 genotypes.
